# Supplementary material for: Effects of Inulin Propionate Ester Incorporated into Palatable Food Products on Appetite and Resting Energy Expenditure: A Randomised Crossover Study
Source: Nutrients. 2019 Apr 16;11(4):861. doi: 10.3390/nu11040861 (PMC6520886; doi:10.3390/nu11040861)
Supplement: Supplementary file 1 [file nutrients-11-00861-s001.pdf]

Table S1. Nutritional information of unsupplemented food products prior to the addition of 10 g inulin or 10 g IPE [1].

	Unit	Bread Roll		Fruit smoothie		Total [2]
		per 100 g	Per 77 g portion	per 100 g	per 250 g portion	per day
Energy	KJ	1348	1032	235	589	1621
	kcal	317	243	55	138	381
Fat	g	4.3	3.3	0.2	0.5	3.8
of which saturates	g	0.8	0.6	0.1	0.1	0.7
Carbohydrate	g	66.1	50.6	13.2	33	83.6
of which sugars	g	4.6	3.5	12.3	30.8	34.3
Fibre	g	3.5	2.7	1.3	3.2	5.9
Protein	g	7.6	5.8	1	2.4	8.2
Salt	g	1.433	1.097	0.002	0.005	1.102

[1] as assessed using Dietplan 6; [2] Total when one portion of bread roll and fruit smoothie eaten per day. All food products were identical prior to the addition of 10 g inulin or 10 g IPE; Abbreviations: IPE, inulin-propionate ester.

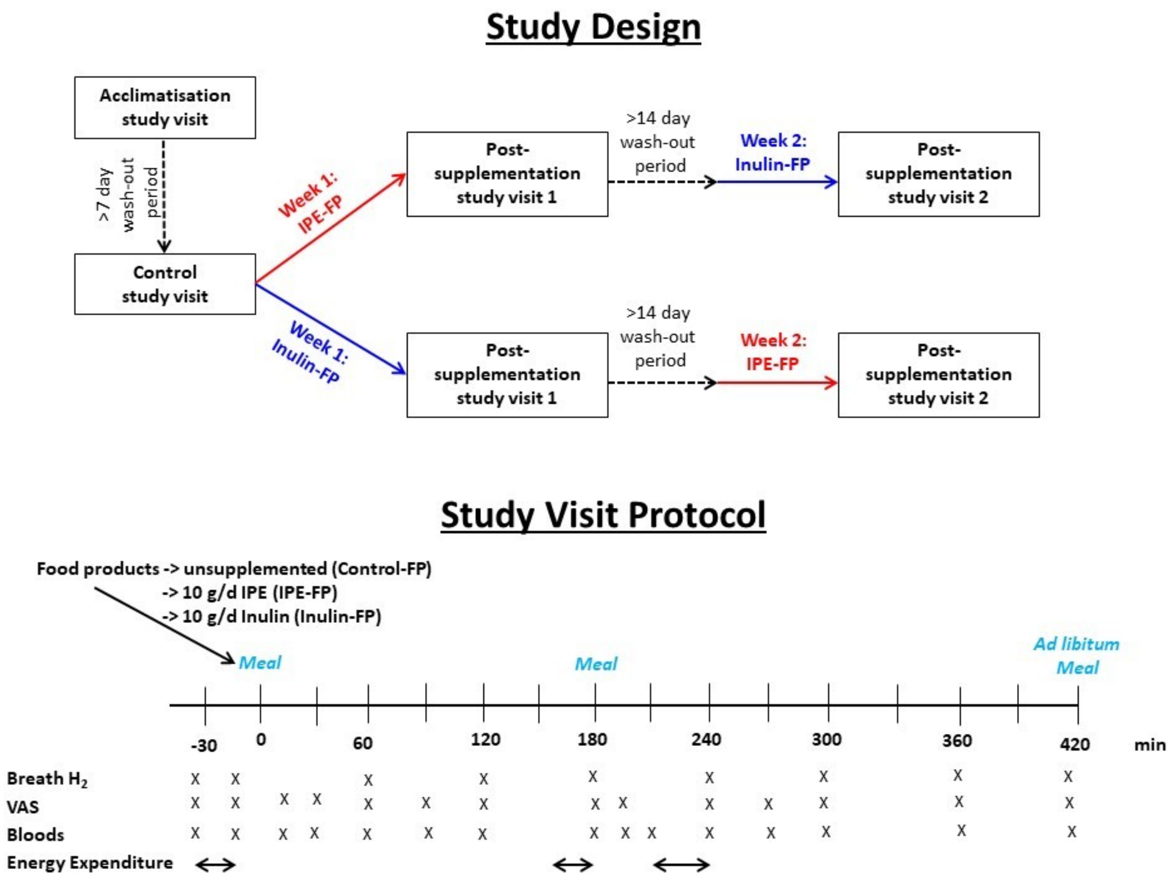

Figure S1. Study overview. Breath H_2, VAS, energy expenditure measurements and blood samples were collected as indicated. Abbreviations: FP, food products; IPE, inulin-propionate ester; VAS, visual analogue scale.

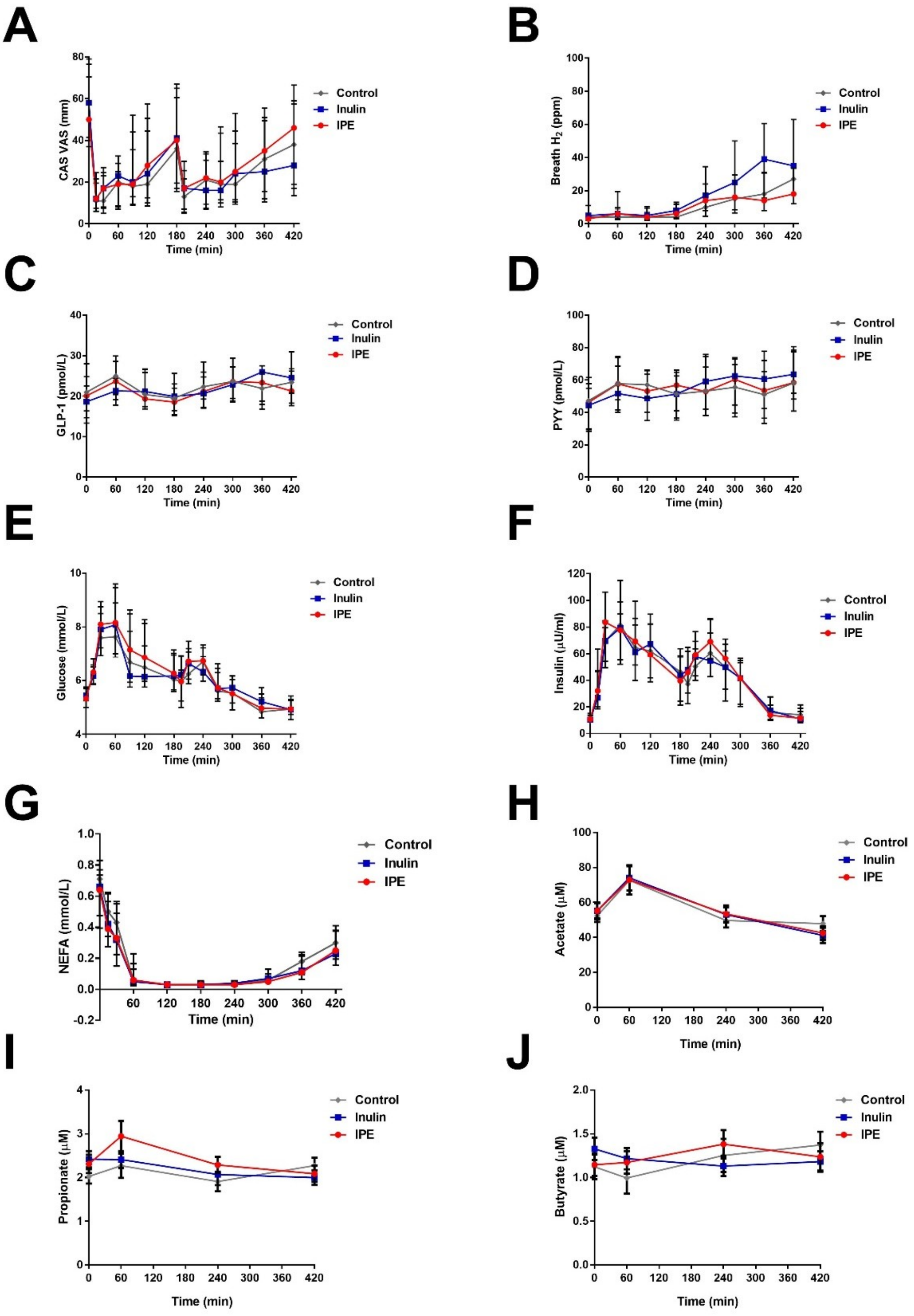

Figure S2. Timecourse data for breath H_2, blood hormones and metabolites following a seven-day supplementation with inulin-FP or IPE-FP compared to control-FP. Data are presented as median [IQR]. Data represent n=21. Abbreviations: CAS, composite appetite score; FP, food products; GLP-1, glucagon-like peptide-1, IPE, inulin-propionate ester; PYY, peptide YY; VAS, visual analogue scale.
